# Divergent Immunomodulation Capacity of Individual Myelin Peptides—Components of Liposomal Therapeutic against Multiple Sclerosis

**DOI:** 10.3389/fimmu.2017.01335

**Published:** 2017-10-16

**Authors:** Vilena V. Ivanova, Svetlana F. Khaiboullina, Marina O. Gomzikova, Ekaterina V. Martynova, André M. Ferreira, Ekaterina E. Garanina, Damir I. Sakhapov, Yakov A. Lomakin, Timur I. Khaibullin, Evgenii V. Granatov, Farit A. Khabirov, Albert A. Rizvanov, Alexander Gabibov, Alexey Belogurov

**Affiliations:** ^1^Institute of Fundamental Medicine and Biology, Kazan Federal University, Kazan, Russia; ^2^Department of Microbiology and Immunology, University of Nevada, Reno, NV, United States; ^3^Shemyakin-Ovchinnikov Institute of Bioorganic Chemistry RAS, Moscow, Russia; ^4^Republican Clinical Neurological Center, Kazan, Russia; ^5^Lomonosov Moscow State University, Moscow, Russia

**Keywords:** myelin basic protein, multiple sclerosis, cytokines/chemokines, liposomal peptide therapeutic, dendritic cells, T helper cells, T regulatory cells, treatment

## Abstract

Multiple sclerosis (MS) is an autoimmune disease characterized by demyelination and consequent neuron injury. Although the pathogenesis of MS is largely unknown, a breach in immune self-tolerance to myelin followed by development of autoreactive encephalitogenic T cells is suggested to play the central role. The myelin basic protein (MBP) is believed to be one of the main targets for autoreactive lymphocytes. Recently, immunodominant MBP peptides encapsulated into the mannosylated liposomes, referred as Xemys, were shown to suppress development of experimental autoimmune encephalomyelitis, a rodent model of MS, and furthermore passed the initial stage of clinical trials. Here, we investigated the role of individual polypeptide components [MBP peptides 46–62 (GH17), 124–139 (GK16), and 147–170 (QR24)] of this liposomal peptide therapeutic in cytokine release and activation of immune cells from MS patients and healthy donors. The overall effects were assessed using peripheral blood mononuclear cells (PBMCs), whereas alterations in antigen-presenting capacities were studied utilizing plasmacytoid dendritic cells (pDCs). Among three MBP-immunodominant peptides, QR24 and GK16 activated leukocytes, while GH17 was characterized by an immunosuppressive effect. Peptides QR24 and GK16 upregulated CD4 over CD8 T cells and induced proliferation of CD25^+^ cells, whereas GH17 decreased the CD4/CD8 T cell ratio and had limited effects on CD25^+^ T cells. Accordingly, components of liposomal peptide therapeutic differed in upregulation of cytokines upon addition to PBMCs and pDCs. Peptide QR24 was evidently more effective in upregulation of pro-inflammatory cytokines, whereas GH17 significantly increased production of IL-10 through treated cells. Altogether, these data suggest a complexity of action of the liposomal peptide therapeutic that does not seem to involve simple helper T cells (Th)-shift but rather the rebalancing of the immune system.

## Introduction

Multiple sclerosis (MS) is an autoimmune disease of unknown etiology affecting 2.5 million people worldwide between the ages of 20 and 50 and is histologically characterized by axonial demyelinization and neuronal death (1). It is believed that loss of the myelin is the result of the appearance of a pool of myelin-reactive lymphocytes (2) occurring due to the breakage of immune tolerance (3). Studies using animal models confirmed the essential role of one of the major components of the myelin sheath, the myelin basic protein (MBP), in MS pathogenesis, as transfer of MBP-sensitized T cells induced experimental autoimmune encephalomyelitis (EAE), a disease in mice similar to MS (4). Fridkis-Hareli et al. identified MBP residues 85–99 as immunodominant epitope (5), and a CD4^+^ T cell clone specific for MBP epitope 87–99 was shown to be capable of EAE induction (6). In addition, Huseby et al. demonstrated that the MBP-specific CD8^+^ T cells can trigger severe EAE (7), suggesting that autoimmune neurodegeneration may be mediated by CD8^+^ lymphocytes as well. Direct evidence of existence of myelin-specific cytotoxic lymphocytes targeting oligodendrocytes *in vitro* has been reported (8), therefore supporting the importance of MBP in the pathogenesis of MS.

At the present time, a number of therapeutics with various mechanisms of action are approved for MS treatment (1) including beta-interferons (IFNs) (9), glatiramer acetate (GA) (10–13), and monoclonal antibodies (14, 15). Historically, MBP and its peptide derivates (16–20) were considered as potential candidates for specific autoantigen immunotherapy (21). In our previous studies, we accomplished epitope profiling of autoantibodies from MS patients and identified several immunodominant epitopes located on the 46–62, 124–139, and 147–170 aa positions of the MBP (22). We have shown that administration of these peptides in native form (23) or a microencapsulated form (24) significantly suppressed EAE development. Recently, completed clinical trials of the liposomal peptide therapeutic, referred as Xemys, representing MBP peptides 46–62, 124–139, and 147–170 entrapped into the small mannosylated monolayer liposomes (25), revealed a decrease in the levels of monocyte chemoattractant protein-1 (MCP-1/CCL2), macrophage inflammatory protein-1 (MIP-1/CCL4), interleukin (IL)-7, and IL-2 in the serum of treated MS patients (26). By contrast, the serum levels of tumor necrosis factor (TNF) were remarkably elevated. A shift in the observed dichotomy of cytokines in response to administration of liposomal peptide therapeutic prompted us to investigate the effect of MBP peptides on cytokine secretion and activation of peripheral blood mononuclear cells (PBMCs) representing leukocytes and plasmacytoid dendritic cells (pDCs) from MS patients and healthy donors (HDs). Collected data will be useful for design of the future clinical trials that could help to estimate the therapeutic efficacy of the liposomal peptide therapeutic.

## Materials and Methods

### Blood Samples

Ten untreated MS subjects (aged 36.5 ± 5.6 years) admitted to the Republican Clinical Neurological Center, Republic of Tatarstan, were enrolled into this study. MS diagnosis was based on clinical presentation and magnetic resonance imaging data. Blood samples (10 mL) were collected from each enrolled MS subject and from 10 healthy individuals. This study was carried out in accordance with the recommendations of local ethics committee of the Republican Clinical Neurological Center with written informed consent from all subjects. All subjects gave written informed consent in accordance with the Declaration of Helsinki. The protocol #16 from 29 October 2015 was approved by the local ethics committee of the Republican Clinical Neurological Center.

### MBP Peptides

Myelin basic protein peptides were synthesized using a solid-phase technique as described by Belogurov et al. (24). Peptide expected mass was confirmed by matrix-assisted laser desorption/ionization–time of flight mass spectrometry. Sequences of MBP peptides are summarized in Table S1 in Supplementary Material. Freeze-dried peptides were stored at −20°C. The concentration of each peptide was determined using the BCA Protein Assay Kit (SigmaAldrich, St. Louis, MO, USA).

### PBMC Separation and pDCs Isolation

Peripheral blood mononuclear cells were separated by Histopaque-1077 (SigmaAldrich, St. Louis, MO, USA) density gradient sedimentation. pDCs were isolated by negative selection using the Plasmacytoid Dendritic Cell Isolation kit (Miltenyi Biotec Inc., Auburn, CA, USA) followed by positive pDC selection according to the manufacturer’s instructions. Separated pDCs were maintained in IMDM supplemented with 10% FBS and IL-3 (10 ng/mL). The purity of pDC culture was confirmed by flow cytometry to be 97%. PBMCs, including pDCs, were maintained in RPMI-1640 complete medium supplemented with 10% FBS.

### Peptide Priming

Peripheral blood mononuclear cells and pDCs were plated into 96-well plates at a density of 5 × 10^3^ cells/well in RPMI-1640 and 2.5 × 10^3^ cells/well in IMDM culture media, respectively. Cells were incubated in the presence of each MBP peptide (10 µg/mL for PBMCs and 5 µg/mL for pDCs; 0.2% DMSO in PBS) at 37°C in a humidified atmosphere of 5% CO_2_. As a negative control, PBS containing 0.2% DMSO was added into the culture. At various time points (0.5, 1, 3, 6, 12, and 24 h), cells and supernatants were collected and stored at −80°C until analysis. For the flow cytometry analysis, cells were collected after 24 h of incubation with each MBP peptide as well as controls.

### Flow Cytometry

Peripheral blood mononuclear cells and pDCs were incubated with antibody conjugates for 20 min in the dark. Antibodies used for flow analysis are summarized in Table S2 in Supplementary Material. At the end of the incubation, cells were washed twice with DPBS (PanEco, Moscow, Russia) and fixed in 4% paraformaldehyde (10 min; 37°C). Cells were washed twice with DPBS (PanEco, Moscow, Russia) and analyzed using the Guava EasyCyte™ 8 HT flow cytometer (Merck, Darmstadt, Germany). Cells expressing surface markers were presented as a percent of positive leukocytes. The percentages of CD4^+^ and CD8^+^ cells were determined within the total leukocyte population (CD45^+^ cells). The percentage of CD25 leukocytes was determined within the CD4^+^ cells.

### Enzyme-Linked Immunosorbent Assay (ELISA)

ELISA kits for measurement of tumor necrosis factor, interferon-α (IFN-α), interferon-γ (IFN-γ), IL-6, IL-8, and IL-10 were purchased from Vektor-BEST (Novosibirsk, Russia). The level of cytokines in supernatants from PBMCs and pDCs was analyzed at selected time points (0.5, 1, 3, 6, 12, and 24 h). The 3,3′,5,5′-tetramethylbenzidine substrate (450 nm) was used to visualize results using a TECAN infinite M200PRO microplate reader (Tecan, Männedorf, Switzerland).

### RNA Isolation and cDNA Synthesis

Total RNA was isolated using TRIzol Reagent (Thermo Fisher Scientific, Waltham, MA, USA) according to the manufacturer’s recommendations. Synthesis of cDNA was carried out using 100 U Maxima Reverse Transcriptase (Thermo Fisher Scientific, Waltham, MA, USA). For cDNA synthesis, an RNA/primer/dNTP mix [100 ng of RNA, 1 µL of random hexamer primers (Litekh, Moscow, Russia), and 1 µL dNTP mix (10 mM)] in total volume of 10 µL was denatured at 65°C for 5 min and chilled quickly to 4°C. cDNA was synthesized by adding 5× RT buffer (Thermo Fisher Scientific, Waltham, MA, USA), 200 U of RevertAid Reverse Transcriptase and 20 U of Ribolock RNAse inhibitor (Thermo Fisher Scientific, Waltham, MA, USA) in a 20-µL volume of the reaction mix. After incubation (10 min, 25°C), the reaction was proceeded for another 30 min at 42°C and finally terminated by heating at 70°C for 10 min.

### Real-time PCR (RT-PCR)

The RT-PCR reaction mix (15 µL) consisted of 0.5 µL cDNA (100 ng), 2.5× Reaction mix B (Syntol, Moscow, Russia), 200 nM of each primer, and 100 nM of probe (Table S3 in Supplementary Material). Amplification parameters were as follows: preheating at 95°C for 3 min followed by 39 cycles: 95°C for 10 s, and 55°C for 15 s. RT-PCR values for each gene were normalized to corresponding values of 18S ribosomal RNA. Standard curves for relative quantitation of the TNF transcripts were generated using 10-fold serial dilutions of cDNA. Values obtained for each gene in control (PBS-treated PBMCs) were considered as 100%. All qPCR reactions were performed in triplicates.

### Statistical Analysis

Data are expressed as means ± SEM. Statistical analysis was performed by factorial ANOVA followed by the least significant means *post hoc* test. Significance was established at a value of *p* < 0.05. All statistical analyses were performed using the GraphPad Prism 6 software.

## Results

### Evaluation of Expression of Surface Activation Markers by PBMCs Treated with MBP Peptides Containing in Liposomal Peptide Therapeutic

**Figure 1:**
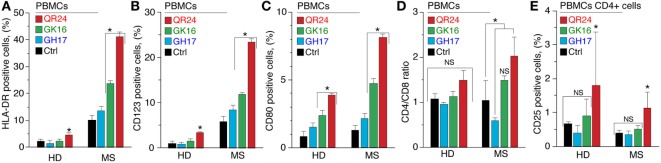
Expression of the cell surface markers by peripheral blood mononuclear cells (PBMCs) exposed to the individual myelin basic protein (MBP) peptides comprising the liposomal peptide therapeutic. The PBMCs from the healthy donors (HDs) and multiple sclerosis (MS) patients were incubated with 10 µg mL^−1^ of MBP peptides GH17 (blue bars), GK16 (green bars), QR24 (red bars), or PBS (black bars) for 24 h. Percentages of human leukocyte antigen (HLA-DR)^+^ **(A)**, CD80^+^ **(B)**, CD123^+^ **(C)**, and CD25^+^ **(E)** cells were further determined by flow cytometry. Data are presented as a percentage of positive cells within the CD45^+^ PBMC population. The percentage of CD25^+^ cells was estimated in the CD4^+^ population. CD4/CD8 ratio **(D)** was estimated as an absolute count of CD4 cells divided by the absolute count of CD8 cells in each sample. Asterisk denotes statistically significant difference with a control. NS, non-significant.

To study the effect of MBP peptides from the liposomal peptide therapeutic on the expression of surface activation markers, purified PBMCs from MS and HDs were incubated with individual peptides GH17, GK16, and QR24 and further stained for human leukocyte antigen (HLA-DR), CD4, CD8, CD25, and CD80 (Figure 1). Expression of HLA-DR and CD123 molecules by PBMCs in response to MBP peptides differed dramatically between HDs and MS patients (Figure 1A). The number of HLA-DR^+^ cells in PBMCs from HDs changed from 2.1% (PBS) to 1.5, 2.3, and 4.6% on incubation with GH17, GK16, and QR24, respectively. Exposure of PBMCs from MS patients to MBP peptides resulted in significantly enhanced proportions of HLA-DR^+^ cells, estimated as 13.6, 23.8, and 41.1% on incubation with GH17, GK16, and QR24, respectively. The number of CD123^+^ cells in PBMCs from HDs and MS patients on incubation with QR24 increased up to 4 and 24%, respectively.

Study of the expression of CD80, a costimulatory signal necessary for T cell activation and survival, on PBMCs in response to the MBP peptide revealed that GK16 and QR24 increased the expression of CD80 on cells from both MS patients and HDs (Figure 1C). Similar to HLA-DR, PBMCs from MS responded more pronouncedly in comparison with HDs: the percentage of CD80^+^ cells increased from ~1 to 4.8–8.1% in MS and 2.4–3.9% in HD in case of exposure to GK16 and QR24, respectively.

We next measured the CD4/CD8 ratio in PBMCs treated by MBP peptides. Peptides QR24 and GK16 increased this ratio to 1.5–2.0, whereas GH17 induced changes in the CD4/CD8 ratio to values less than 1.0 in PBMCs from MS (Figure 1D). Changes in the CD4/CD8 ratio in PBMCs from HDs as a response to the peptide addition were generally statistically insignificant. We next examined the effect of MBP peptides on the CD25^+^ population of PBMCs. Incubation of PBMCs with GK16 and GH17 did not change levels of CD25^+^ cells, whereas peptide QR24 increased the number of CD25^+^ cells as compared with control incubation with PBS (Figure 1E).

### Analysis of Cytokine Production by PBMCs Treated with Peptides from Liposomal Peptide Therapeutic

**Figure 2:**
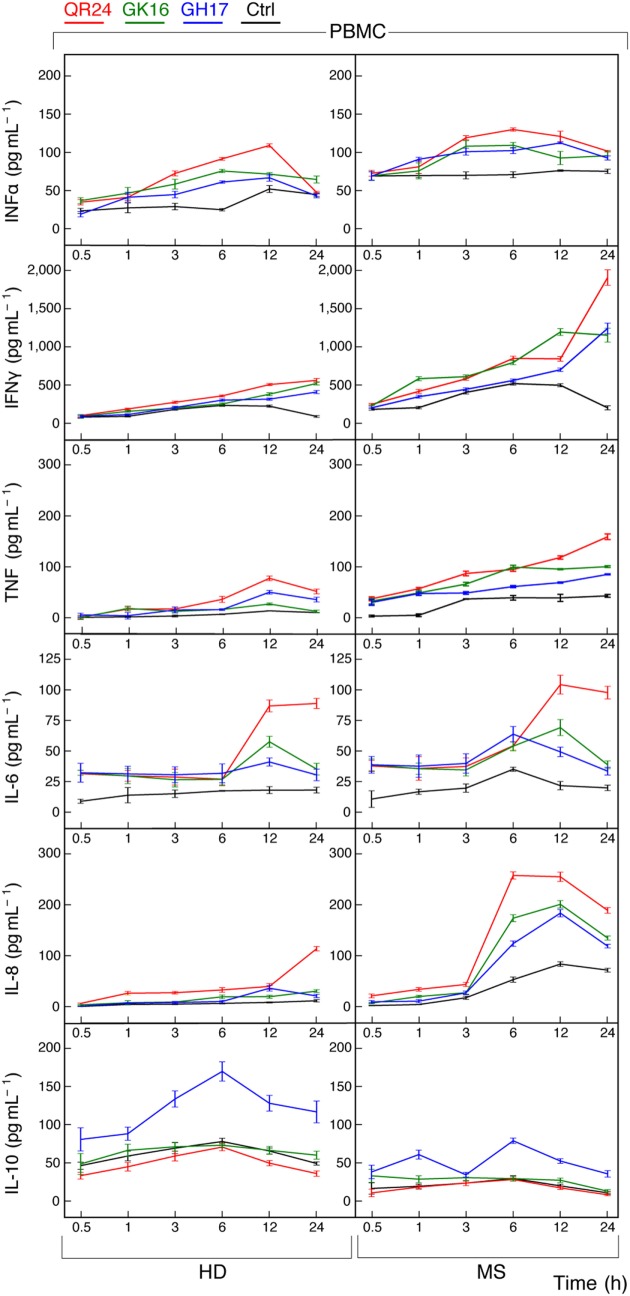
Cytokine production by peripheral blood mononuclear cells (PBMCs) treated by individual myelin basic protein (MBP) peptides comprising the liposomal peptide therapeutic. The PBMCs from the healthy donors (HDs, left panels) and multiple sclerosis (MS) patients (right panels) were incubated with 10 µg mL^−1^ of MBP peptides GH17 (blue curves), GK16 (green curves), QR24 (red curves), or PBS (black curves). Levels of cytokines [from top to bottom, interferon (IFN)-α, IFN-γ, tumor necrosis factor (TNF), interleukin (IL)-6, IL-8, and IL-10] in the supernatants of the PBMCs were determined at indicated time points (0.5, 1, 3, 6, 12, and 24 h).

Both Th1 and Th2 helper T cell phenotypes are activated in MS (27, 28). To elucidate whether MBP peptides can shift the balance toward either Th1 or Th2, we measured cytokines released by PBMCs from HD and MS patients treated by MBP peptides. Supernatants from cultured cells were collected at various time points and further levels of IL-6, IL-8, IL-10, IFN-α, IFN-γ, and TNF were measured by ELISA (Figure 2).

The basal level of IFN-α in PBMCs from MS patients was higher compared with that of PBMCs from HDs. Concentration of IFN-α in response to all MBP peptides was changed in a top-shaped mode and increased by approximately two times after 6–12 h in PBMCs from MS patients and HDs. A significant increase in the concentration of IFN-γ was observed only after 24 h of PBMC stimulation by any one of the three MBP peptides, herewith the resulting level of IFNγ was two to four times higher in PBMCs from MS patients. Similarly, the level of TNF after 24 h of incubation with MBP peptides was two to three times higher in PBMCs from MS patients in comparison with the HDs. A statistically significant fivefold increase of IL-6 was detected in PBMCs treated by QR24 regardless the origin of the cells. Levels of IL-8 were elevated in PBMCs from MS patients treated by any one of the MBP peptides, whereas only the peptide QR24 increased level of IL-8 in PBMCs from HDs. Interestingly, from the three MBP peptides only GH17 increased IL-10 production in PBMCs regardless of their origin; the level of IL-10 in GH17-treated PBMCs from HD was two times higher than those in cells from MS patients.

**Figure 3:**
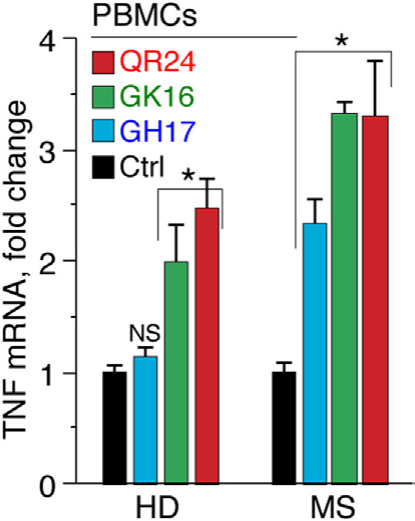
Level of mRNA-encoding tumor necrosis factor (TNF) in peripheral blood mononuclear cells (PBMCs) in response to incubation with individual myelin basic protein (MBP) peptides comprising the liposomal peptide therapeutic. The PBMCs from the healthy donors (HD) and multiple sclerosis (MS) patients were incubated with 10 µg mL^−1^ of MBP peptides GH17 (blue bars), GK16 (green bars), QR24 (red bars), or PBS (black bars) for 12 h. Further levels of mRNA-encoding TNF were measured by qPCR. All measurements were performed in triplicates, and values were normalized to corresponding values of 18S ribosomal RNA.

Recently, we have shown that the serum levels of TNF were markedly elevated in MS patients treated by liposomal peptide therapeutic (26). To corroborate our data on TNF activation in leukocytes treated with MBP peptides, levels of TNF mRNA were analyzed using RT-PCR (Figure 3). Obtained data suggest that QR24 and GK16 increased the level of mRNA-encoding TNF in PBMCs from HD and MS patients, whereas GH17 upregulated levels of TNF mRNA only in PBMCs from MS patients.

### Analysis of Expression of Surface Activation Markers and Cytokine Release by pDCs Exposed to MBP Peptides from Liposomal Peptide Therapeutic

**Figure 4:**
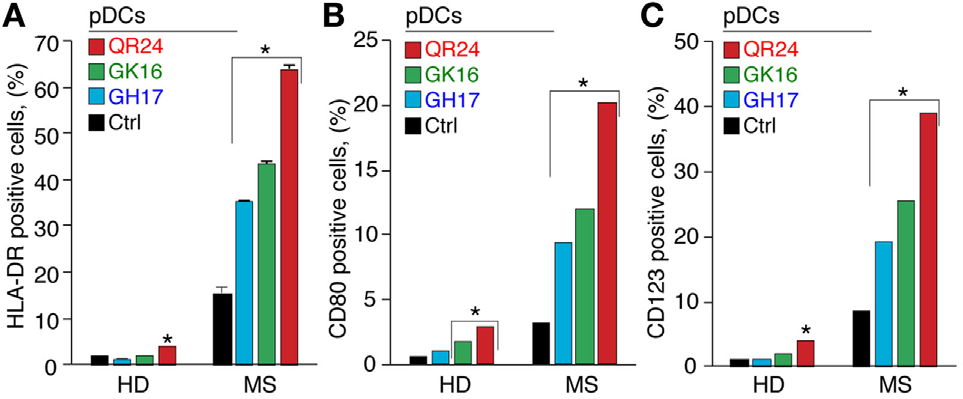
Expression of the cell surface markers by plasmacytoid dendritic cells (pDCs) exposed to the individual myelin basic protein (MBP) peptides comprising the liposomal peptide therapeutic. The pDCs from the healthy donors (HD) and multiple sclerosis (MS) patients were incubated with 5 µg mL^−1^ of MBP peptides GH17 (blue bars), GK16 (green bars), QR24 (red bars), or PBS (black bars) for 24 h. Percentages of human leukocyte antigen (HLA-DR)^+^ **(A)**, CD80^+^ **(B)**, and CD123^+^ **(C)** cells were further determined by flow cytometry. Data are presented as a percentage of positive cells within the CD45^+^ PBMC population. Asterisk denotes a statistically significant difference with the control. NS, non-significant.

The pDCs represent a minor population of leukocytes, playing pivotal role in regulation of self-tolerance (29). Therefore, the role of pDCs in pathogenesis of autoimmune diseases is currently under careful consideration (30). To elucidate the effect of MBP peptides comprising the liposomal peptide therapeutic on pDCs, expression of surface activation markers and cytokine production was analyzed in MS and HD pDCs, incubated with GH17, GK16, and QR24. The pDCs treated with each of three peptides significantly increased the expression of HLA-DR, CD80, and CD123 (Figure 4). Notably, there was a 5- to 10-fold difference in surface activation marker upregulation between MS and HD pDCs, where peptide QR24 induced the most pronounced effect on pDCs.

**Figure 5:**
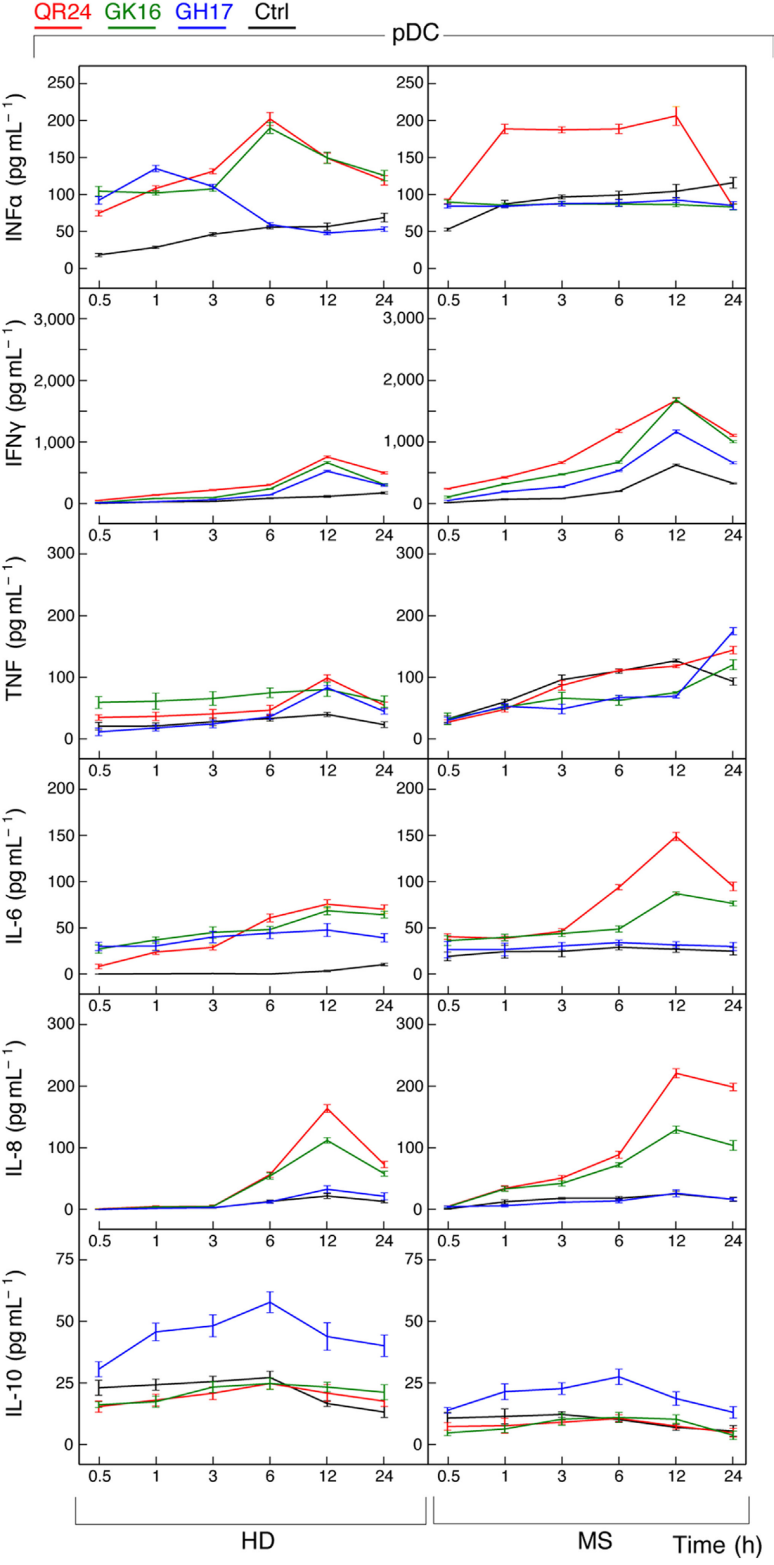
Cytokine production by plasmacytoid dendritic cells (pDCs) treated by individual myelin basic protein (MBP) peptides comprising the liposomal peptide therapeutic. The pDCs from the healthy donors (HDs, left panels) and multiple sclerosis (MS) patients (right panels) were incubated with 5 µg mL^−1^ of MBP peptides GH17 (blue curves), GK16 (green curves), QR24 (red curves), or PBS (black curves). Levels of cytokines [from top to bottom, IFN-α, IFN-γ, tumor necrosis factor (TNF), IL-6, IL-8, and IL-10] in the supernatants from the peripheral blood mononuclear cells were determined at indicated time points (0.5, 1, 3, 6, 12, and 24 h).

Profile of cytokines release by pDCs exposed to the MBP peptides was in general similar to those data observed in case of PBMCs (Figure 5). Incubation with each MBP peptide caused an early increase (occurring in less than in 30 min) in IFN-α production by pDCs from HDs that after 6 h gradually declined. By contrast, only QR24 peptide upregulated IFN-α production in MS pDCs, while the cytokine levels remained unchanged when cells were incubated with GK16 and GH17. The level of IFN-γ after 12 h of incubation with each of three MBP peptides increased from twofold to threefold in pDCs from both, HD and MS patients. There was no statistically significant difference in the concentration of TNF in pDCs from MS patients treated by MBP peptides, whereas a twofold to threefold increase in TNF level was observed in pDCs from HDs exposed to each of MBP peptides. The level of IL-6 was increased in pDCs from HDs in response to incubation with each of the MBP peptides, while after 12 h of incubation only GK16 and QR24 enhanced release of IL-6 by pDCs from MS patients by threefold to sixfold. Concentration of IL-8 was increased 10–20 times in the case of incubation of pDCs from HDs and MS patients with GK16 and QR24 but not GH17. By contrast, the concentration of IL-10 was increased exclusively in pDCs exposed to GH17, whereas neither QR24 nor GK16 was able change the levels of this cytokine.

## Discussion

The pathogenesis of autoimmune disorders is mainly caused by break of tolerance to self-antigens (31), thus, its restoration may prevent or even reverse the autoimmune pathology. This assumption can be applied to MS pathogenesis as well, as administration of MBP increased the resistance of animals to EAE, an MS animal model (32). In the line with this reasoning, symptoms of EAE could be reversed by the administration of MBP-tolerized T cells (6). Being encapsulated into the mannosylated liposomes in case of *in vivo* administration MBP peptides will be mainly delivered into the CD206^+^ cells, namely, antigen-presenting cells (APC) like dendritic cells (24). Therefore, it is unlikely that CD4^+^ or T regulatory cells (Tregs) will be granted any access to these peptides in a “free” form. More realistic scenario is that dendritic cells will present these peptides to other immune cells. That was the reason for us to study activation of two cell populations from either healthy donors or MS patients: (i) PBMCs that *a priori* will contain APC and (ii) isolated pDCs to more specifically understand the immunomodulation capacity of MBP peptides.

The effect of GH17 peptide (MBP46–62) was distinct from that of the remaining two peptides QR24 (MBP147–170) and GK16 (MBP124–139), and it was characterized by decreased number of cells expressing surface activation markers. Analysis of the CD4/CD8 lymphocyte ratio revealed that peptides QR24 and GK16 shifted this balance toward CD4 cells, whereas peptide GH17 reversed it to the CD8 cells. In addition, the GH17 peptide was more effective in activation of expression of anti-inflammatory cytokine IL-10 but not pro-inflammatory cytokines. By contrast, peptides QR24 and GK16 upregulated the expression of pro-inflammatory cytokines, whereas the effect of these peptides on the release of IL-10 was absent. Therefore, similar to GA (33, 34), the peptide GH17 appeared to shift the Th1/Th2 balance to Th2, whereas peptides QR24 and GK16, by contrast, shifted this balance toward Th1.

Peptide GH17 had at a glance paradoxical effect on PBMCs and pDCs, namely, it enhanced release of IL-10 and at the same time increased level of mRNA coding for TNF. Detailed analysis of the curves representing concentration of TNF and IL-10 in culture medium from PBMCs and pDC incubated with GH17 revealed that concentration of IL-10 raised first 12 h and began to decrease exactly when concentration of TNF started to increase (Figure S1 in Supplementary Material). This observation is in line with previously reported data suggesting that IL-10 negatively regulates production of TNF (35) by destabilization of TNF-encoding mRNA (36). Therefore, we suggest that expression of IL-10 and increase of TNF mRNA leading to release of TNF by cells exposed to GH17 are not parallel but time-resolved processes.

**Figure 6:**
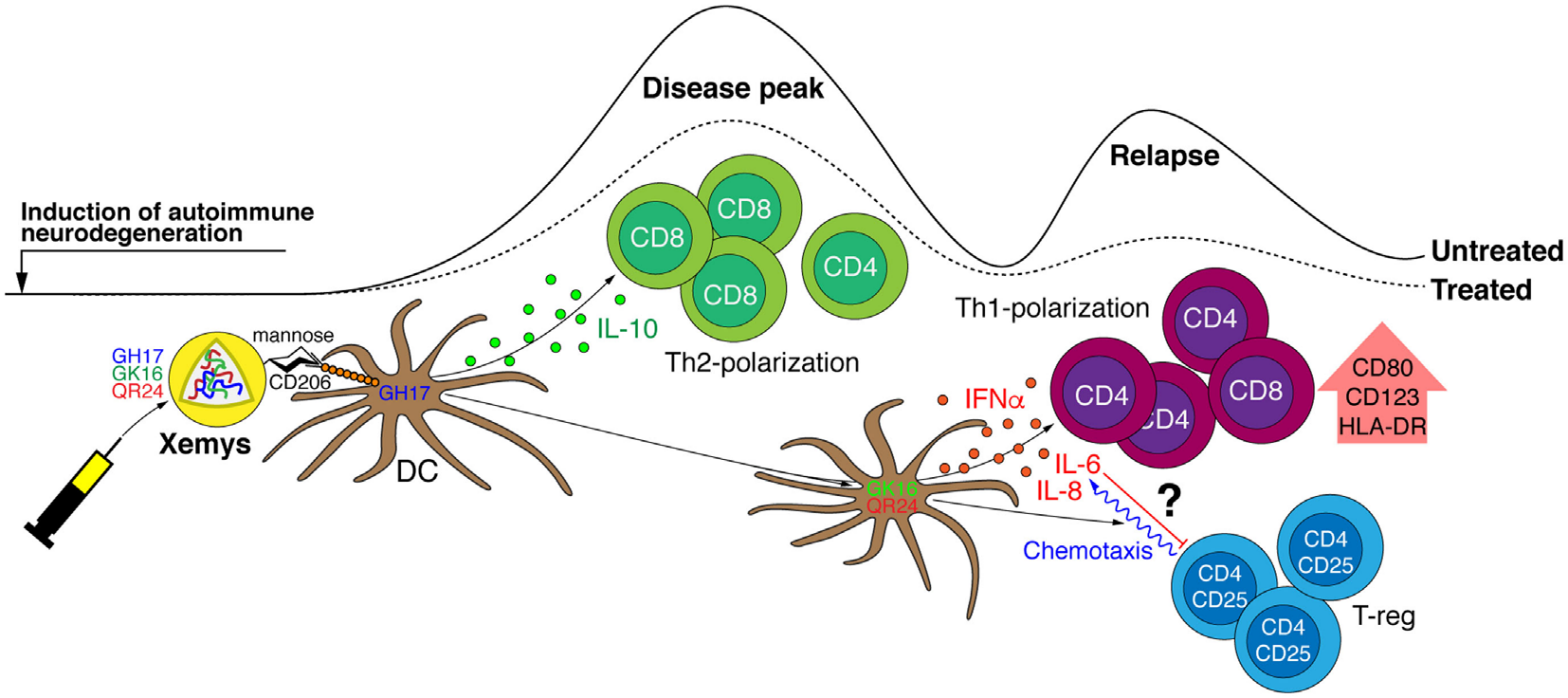
Fine tuning of the immune system by individual peptide components of the liposomal peptide therapeutic. Liposome-encapsulated myelin peptides GH17, GK16, and QR24 are proposed to be mainly trapped by dendritic cells through CD206 receptors due to the exposed mannose residues on the surface of liposomes. It seems that GH17 has a limited effect on leukocyte activation, rebalances T cells to being CD8^+^, and induces production of anti-inflammatory cytokine IL-10 that may significantly contribute to the amelioration of the first disease attack. By contrast, QR24 and GK16, which are effective mainly in preventing a second wave of exacerbation, induce the release of pro-inflammatory cytokines, shifting the CD4/CD8 ratio to CD4 T cells and promoting proliferation of CD4^+^CD25^+^ lymphocytes, which are important for maintaining further immune tolerance.

Interestingly, studied MBP peptides differed in their ability to inhibit EAE, as has been shown in our previous report (24). For example, peptides QR24 and GK16 were effective mainly in preventing a second wave of EAE exacerbation, while GH17 was the most beneficial in reducing the maximal disease score during the first attack. We propose that the varying effects of MBP peptides on EAE onset and progression could be directly associated with differences in their capabilities to activate PBMCs/pDCs and induce cytokine production (Figure 6). The GH17 peptide showed a limited effect on leukocyte activation and had unique ability to upregulate anti-inflammatory cytokine IL-10, which may significantly contribute to the amelioration of the first disease attack. Two other peptides, QR24 and GK16, which reduced the relapse of EAE, showed a more pronounced pattern of PBMC/pDC activation and release of pro-inflammatory cytokine production. One may suggest that “resetting” the immune system after the disease onset could be beneficial in amelioration of the relapse.

Another important point observed herein suggests that MBP peptide QR24 is capable of promoting proliferation of CD4^+^CD25^+^ Tregs, which are important for maintaining immune tolerance during therapeutic interventions (37), and therefore may suppress disease relapse. Interestingly, elevation of CD25^+^ cells was more pronounced in PBMCs from HDs versus MS, suggesting possible impairment of their regulatory function in MS patients. It should be mentioned that overproduction of IL-6 by QR24-treated cells should in principle inhibit conversion of T cells into the regulatory subset (38) and significantly impair function of Tregs themselves (39). On the other hand, IL-6 and IL-8 may attract Tregs as shown for the immunoescaping tumors (40). Further studies should resolve such dualistic effect of MBP peptide QR24.

Our data for the first time uncover the effect of MBP peptides from liposomal peptide therapeutic on pDC activation and cytokine release by these cells. The pDCs represent a minor population of circulating leukocytes (41) producing copious amounts of IFN-α (42). Their main function is to establish self-tolerance to nucleic acids and to provide the first line of defense against viruses and microbes (43). Recent studies have shown that pDCs play a central role in the pathogenesis of autoimmune diseases such as systemic lupus erythematosus (4), and recently, the role of pDCs in MS development was also suggested as important (44). We have shown that pDCs from MS patients respond to MBP peptides more vigorously as compared with those from HDs, indicating prior exposure of pDCs from MS patients to MBP epitopes. Our data suggest that, similar to PBMCs, MBP peptides QR24 and GK16 have the potential to activate pDCs and trigger the production of pro-inflammatory cytokines. On the contrary, GH17 is characterized by a limited effect on expression of surface activation markers and production of pro-inflammatory cytokines. Importantly, the restricted ability of the pDCs from MS patients to produce IL-10 in response to the GH17, in contrast to the pDCs from HDs, may significantly restrict their capabilities in tolerance reinforcement, resulting in disease triggering and development.

In conclusion, we suggest that individual peptidyl components of liposomal peptide therapeutic act bidirectionally, and therefore the overall effect of this formulation seems to be the fine tuning of the immune system rather than simplified unspecific suppression of immune cells or a Th1/Th2 shift.

## Ethics Statement

This study was carried out in accordance with the recommendations of local ethics committee of the Republican Clinical Neurological Center with written informed consent from all subjects. All subjects gave written informed consent in accordance with the Declaration of Helsinki. The protocol #16 from 29 October 2015 was approved by the local ethics committee of the Republican Clinical Neurological Center.

## Author Contributions

MG collected flow cytometry data; EM performed cytokine profile analysis; AF and YL were responsible for statistical analysis and graphic design; SK, AG, and AB designed the research; FK, DS, EEG, and VI performed data collection; AR made intellectual contributions to data analysis, discussion, and coordination of the research team; EVG undertook serum sample acquisition and patient data management; TK participated in MS diagnosis and sample collection; SK, AG, and AB analyzed data and wrote the paper.

## Conflict of Interest Statement

The authors declare that the research was conducted in the absence of any commercial or financial relationships that could be construed as a potential conflict of interest.
