# Supplementary material for: Divergent Immunomodulation Capacity of Individual Myelin Peptides—Components of Liposomal Therapeutic against Multiple Sclerosis
Source: Front Immunol. 2017 Oct 16;8:1335. doi: 10.3389/fimmu.2017.01335 (PMC5650689; doi:10.3389/fimmu.2017.01335)
Supplement: Supplementary file 1 [file Presentation_1.PDF]

Supplemental information

Divergent immunodomodulation capacity of individual myelin peptides – components of liposomal therapeutic against Multiple Sclerosis

by Ivanova et al.

Supplemental Information includes three Tables and one Supplementary Figure

Supplementary Table 1. Amino acid sequence of MBP peptides

peptides	*Peptide sequences*	*AA position*
GH17	GGDRGAPKRGSGKDSHH	46-62
GK16	GFGYGGRASDYKSAHK	124-139
QR24	QGTLSKIFKLGGRDSRSGSPMARR	147-170

Supplementary Table 2. List of antibodies used in this study for flow cytometry

**Markers**	**Description**	**Manufacturer**
CD80	Anti-human CD80 (2D10) PE	BioLegend
HLA-DR	Anti-human HLA-DR FITC	Beckman Coulter
CD303	Anti-human CD303 (BDCA-2) FITC	BioLegend
CD123	Anti-human CD123(6H6) APC	BioLegend
CD4	Anti-human CD4 FITC	BD Bioscience
CD25	CD25 PE	BD Bioscience
CD3	PerCPCy™5.5	BD Bioscience
CD8	Anti-human CD8 alpha antibody, FITC	Thermo Fisher Scientific

Supplementary Table 3. Primers and probes sequences utilized for cytokine gene expression analysis

**Primer/probe name**	**Nucleotide Sequence (5`-3`)**
TNF-α probe	[FAM]AGGCGCCACCACGCTCTTCT[BH1]
rmh18S-probe	[HEX]ACCGCGCAAGACGGACCAG[BH2]
TNF-α-F	CCTCTTCTCCTTCCTGATCG
TNF-α-R	ATCACTCCAAAGTGCAGCAG
18S-F	GCCGCTAGAGGTGAAATTCTTG
18S-R	CATTCTTGGCAAATGCTTTCG

Supplementary Figure 1

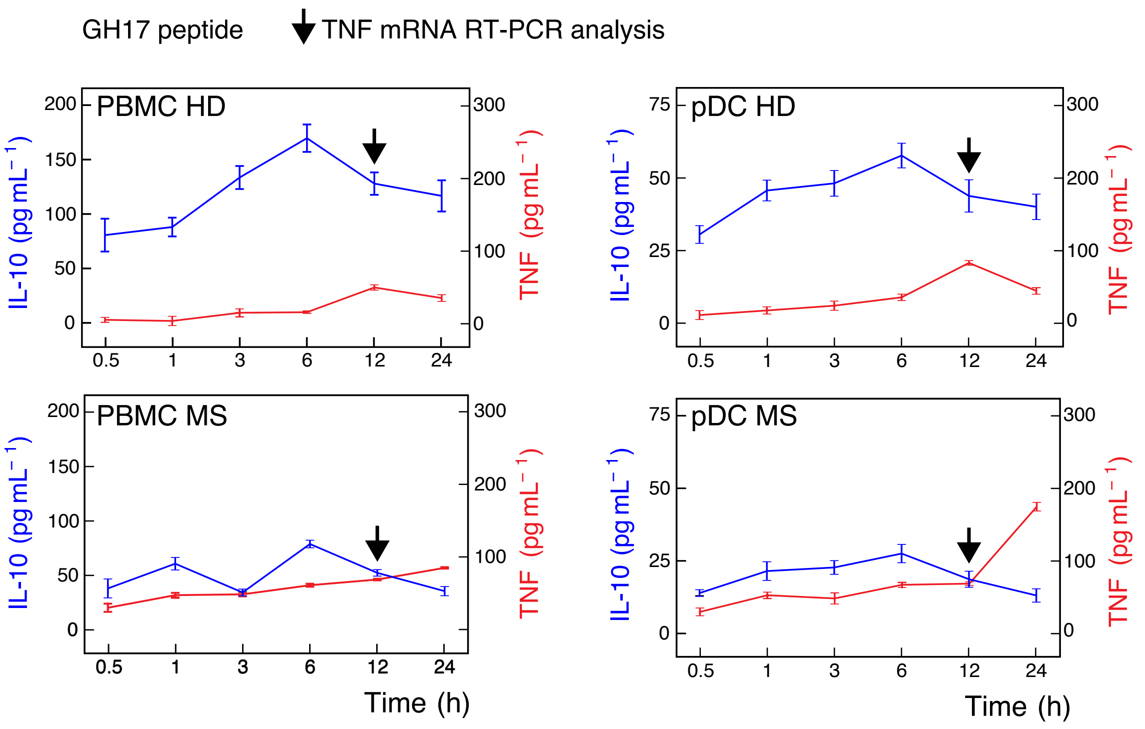

Supplementary Figure 1. Cytokine production by PBMCs and pDCs treated by GH17 peptide. The PBMCs and pDCs from the healthy donors (HD) and multiple sclerosis (MS) patients were incubated with 10 μg/mL of MBP peptide GH17. Levels of tumor necrosis factor (TNF, red curves) and IL-10 (blue curves) in the cell supernatants were determined at indicated time points (0.5, 1, 3, 6, 12 and 24 h). Arrow indicates time point of RT-PCR analysis.
